# QTL Mapping and Data Mining to Identify Genes Associated with Soybean Epicotyl Length Using Cultivated Soybean and Wild Soybean

**DOI:** 10.3390/ijms25063296

**Published:** 2024-03-14

**Authors:** Lin Chen, Shengnan Ma, Fuxin Li, Lanxin Li, Wenjun Yu, Lin Yu, Chunshuang Tang, Chunyan Liu, Dawei Xin, Qingshan Chen, Jinhui Wang

**Affiliations:** 1 National Key Laboratory of Smart Farm Technology and System, Key Laboratory of Soybean Biology in Chinese Ministry of Education, College of Agriculture, Northeast Agricultural University, Harbin 150030, China; venguamecl@163.com (L.C.); mashengnan34@163.com (S.M.); lifuxin202310@163.com (F.L.); lilanxin0530@163.com (L.L.); yuwenjun0825@163.com (W.Y.); cyliucn@neau.edu.cn (C.L.); dwxin@neau.edu.cn (D.X.); 2 Crop Development Research Institute, Heilongjiang Academy of Land Reclamation Sciences, Harbin 150038, China; nkyulin@sina.com (L.Y.); 13644546046@163.com (C.T.)

**Keywords:** soybean, chromosome segment substitution line, epicotyl length, QTL, RNA-seq

## Abstract

Soybean (*Glycine max*) plants first emerged in China, and they have since been established as an economically important oil crop and a major source of daily protein for individuals throughout the world. Seed emergence height is the first factor that ensures seedling adaptability to field management practices, and it is closely related to epicotyl length. In the present study, the Suinong 14 and ZYD00006 soybean lines were used as parents to construct chromosome segment substitution lines (CSSLs) for quantitative trait loci (QTL) identification. Seven QTLs were identified using two years of epicotyl length measurement data. The insertion region of the ZYD00006 fragment was identified through whole genome resequencing, with candidate gene screening and validation being performed through RNA-Seq and qPCR, and *Glyma.08G142400* was ultimately selected as an epicotyl length-related gene. Through combined analyses of phenotypic data from the study population, *Glyma.08G142400* expression was found to be elevated in those varieties exhibiting longer epicotyl length. Haplotype data analyses revealed that epicotyl data were consistent with haplotype typing. In summary, the QTLs found to be associated with the epicotyl length identified herein provide a valuable foundation for future molecular marker-assisted breeding efforts aimed at improving soybean emergence height in the field, with the *Glyma.08G142400* gene serving as a regulator of epicotyl length, offering new insight into the mechanisms that govern epicotyl development.

## 1. Introduction

Soybean (*Glycine max*) originated in China, but it has emerged as an economically important cash crop and food source serving as a major source of protein and edible oil. China remains one of the largest global consumers of soybeans, underscoring a need to increase soybean production efforts [1]. The process of germination is an essential step that ultimately determines whether a seed is able to grow following seeding, with hypocotyl and radicle development status governing soybean seed germination. The epicotyl is a component of the seed embryo, which is the portion between the cotyledon and the opposite true leaf following germination [2]. Epicotyl length (EL) can also impact the height to which plant seedlings grow in the field, as varieties with a shorter EL can be readily covered by soil following the mechanical weeding of the field at the seedling stage, contributing to yield losses [3].

EL is a quantitative trait that is under the control of a limited number of major effector genes and several polygenes, with final EL being sensitive to both genotypic and environmental factors [4,5]. While EL has long been regarded as an important trait in the context of soybean breeding, efforts to breed soybean varieties with adequate EL through traditional selection strategies have been largely inadequate such that there is an urgent need for the development of soybean varieties with better-optimized EL. In 2008, Nogueira et al. included EL and hypocotyl length among soybean characteristics that can be used to determine whether or not a given variety meets the appropriate planting requirements [6]. The majority of these traits have undergone some degree of optimization over the course of the domestication of wild soybeans, including EL [7]. Quantitative trait loci (QTL) are genomic regions associated with the control of certain quantitative traits. In order to identify the relevant genes within these loci, researchers routinely construct populations for QTL mapping [8]. Kaga et al. performed QTL mapping through the use of a cultivated adzuki bean cultivar and the F2:3 generation of the wild bean population as parents, leading to the identification of two QTLs (LG1, LG9) that control EL [7]. Isemura et al. further utilized backcross populations derived from wild beans and Japanese adzuki bean cultivars to identify three QTLs (LG1-1, LG1-2, LG2) [9]. A limited number of hypocotyl development-related QTLs have been reported in soybean, with Liang et al. having employed linkage analysis to identify four soybean hypocotyl-related QTLs [10]. In 2022, Hong et al. reported the first known EL-related QTLs in soybean, collecting 951 soybean germplasm resources from across the globe and utilizing a three-variance component mixed linear model framework (3VmrMLM) to explore the relationship between EL phenotypes and single nucleotide polymorphisms (SNPs) in the genome. In total, 180 EL-related QTNs and QTN-by-environment interactions (QEIs) were detected, with further gene mining ultimately leading to the prediction of 10 seed germination- and epicotyl development-related genes, offering a valuable scientific foundation for further epicotyl research [2]. In the whole process of plant growth and development, transcription factors, as widely existing regulatory proteins in plants, are involved in almost every key stage [11]. At present, the widely reported transcription factor family includes MYB, bHLH, NAC, WRKY, AP2, and so on [12,13,14,15,16]. These transcription factors are involved in the development of different plant tissues and organs during the regulation of plant seedling morphogenesis. For example, MYB112 and WRKY family members have been confirmed to be involved in the regulation of light morphogenesis. Under regular light/dark conditions, MYB112 interacts with phytochrome interacting factor (PIF4), enhancing the transcriptional activity of PIF4, promoting the transmission of auxin-related signals, and then affecting the hypocotyl length (HL) [17]. Under normal sunlight, AtWRKY36 inhibited *AtHY5* transcription by binding to the *AtHY5* promoter region to promote hypocotyl elongation [18]. In addition, bHLH48 and bHLH60, two members of bHLH family, could co-regulate the elongation of Arabidopsis hypocotyls with PIF7 [19]. AP2 is a star transcription factor family that regulates plant growth and development. GmCRF4a, a member of the AP2 family, has been confirmed to play a role in auxin synthesis and in the regulation of soybean plant height. In phenotypic analyses, they found that GmCRF4a overexpression plants presented with increases in EL and HL. Subsequent histological analyses revealed the ability of GmCRF4a to promote epicotyl elongation through increases in cell length [20].

Compared with wild soybean, cultivated soybeans have much lower genetic diversity and higher levels of linkage disequilibrium during domestication. Wild soybeans are the closest wild relatives of cultivated soybeans, have greater allelic diversity than cultivated soybeans, and no reproductive isolation has been shown between cultivated and wild soybeans. This makes wild soybean a promising germplasm whose genetic diversity can be used to improve cultivated soybean traits by gene introgression and the exchange of wild soybeans [21,22]. The present study was developed with the goal of identifying EL-related genes in the soybean genome. To that end, a CSSL population was constructed using two soybean varieties, including one with a long EL (Suinong14) and a wild variety with a short EL (ZYD00006), as parents. The resultant CSSLs were then leveraged to identify EL-related QTLs through a combination of QTL localization, RNA-seq, and chromosome insertion analyses. Candidate genes within these QTL intervals were then identified and validated via qPCR and haplotype analyses.

## 2. Results

### 2.1. Analyses of Epicotyl Length

**Figure 1:**
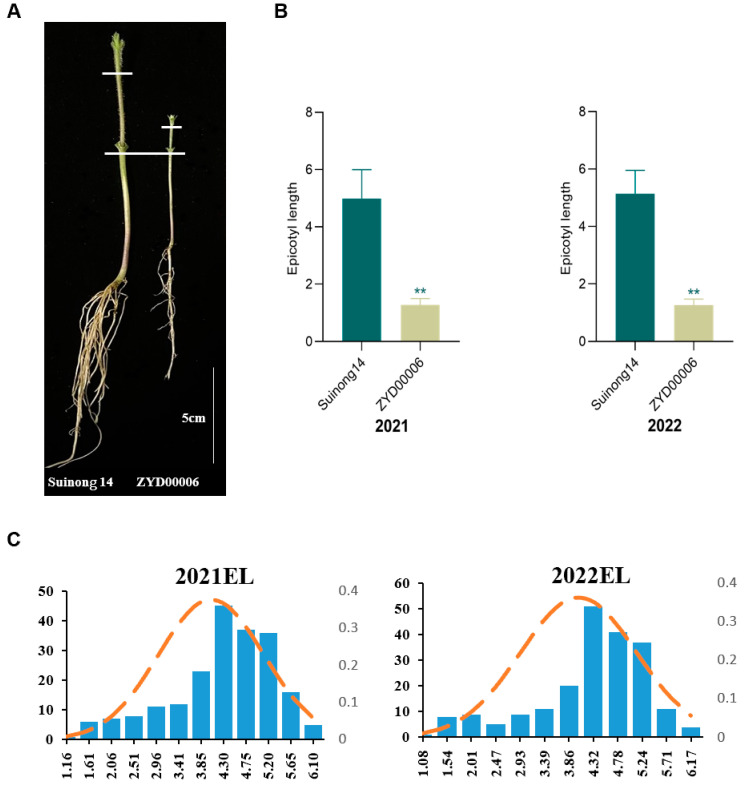
Analyses of the EL of Suinong14, ZYD00006, and CSSL populations. (**A**) Suinong14 and ZYD00006 epicotyl phenotypes. (**B**) The EL of the Suinong14 and ZYD00006 varieties was measured three times. Data were compared with Student’s *t*-tests (** *p* < 0.01). (**C**) EL frequency distributions for the CSSL population in 2021 and 2022.

By analyzing germplasm resources planted during the sowing period, the improved Suinong14 soybean variety was found to exhibit a superior emergence height as compared to the wild ZYD00006 variety, with higher survival rates for the Suinong14 seedlings during the weeding period relative to ZYD00006. This advantage was posited to be attributable to EL, and repeated analyses of the EL of these plants, measured as the distance from the cotyledons to the true leaves, confirmed that Suinong14 plants exhibited an EL significantly longer than that of ZYD00006 plants (Figure 1A,B). Given the significant differences in EL between these two varieties, CSSLs were prepared by crossing the parental Suinong14 and ZYD00006 varieties and backcrossing the resultant offspring. Laboratory EL measurement experiments were conducted on CSSLs harvested in the field across two years (2021 and 2022). The survey data of other agronomic traits in the same year are shown in Table S8. The values ranged from 1.16 cm to 6.10 cm in 2021, and from 1.08 cm to 6.17 cm in 2022, with the EL values of the parents similarly falling within this range (Table 1). As these EL values were normally distributed, the population was deemed suitable for the identification of epicotyl development-related QTLs (Figure 1C, Table S2).

### 2.2. Identification of EL-Related QTLs in CSSL Population

EL data for these CSSL populations collected in 2021 and 2022 were next analyzed using WinQTL Cartographer via the compound interval mapping (CIM) method, setting the detection step to 1.0 cM for 1000 permutation tests. In total, seven valid EL-associated QTLs were identified via screening through this approach (*p*-value < 0.05 and LOD scores > 3.0) (Table 2). When the EL-related QTLs identified for these CSSL populations in both years were compared, a QTL locus on chromosome 8 (LGs A2) was co-located in both years. This QTL interval on chromosome 8 was thus selected for further evaluation.

### 2.3. Determination of Candidate Intervals Based on Chromosomal Insertions

**Figure 2:**
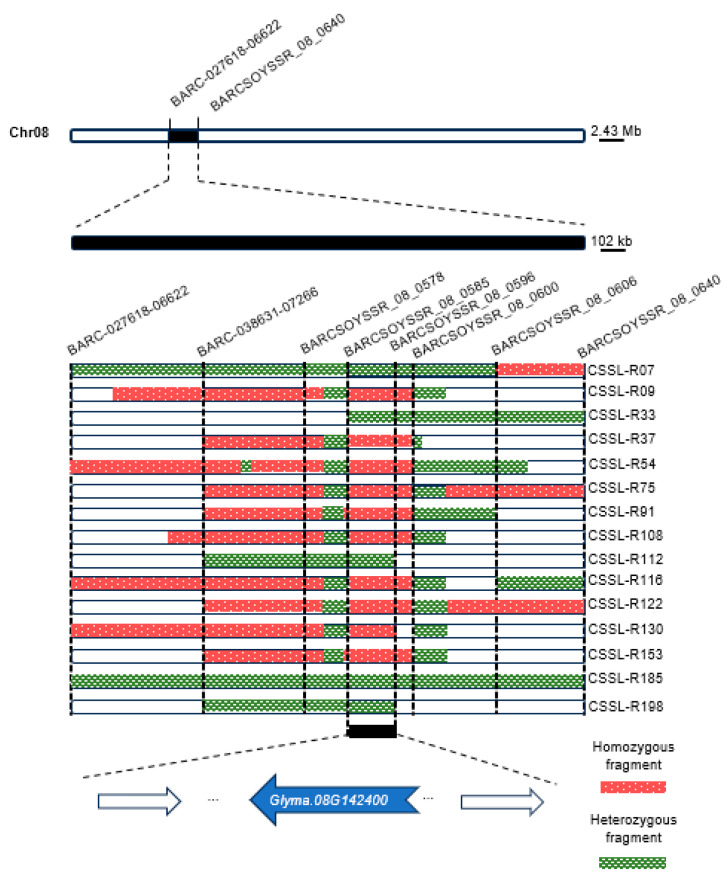
Phenotypic fine mapping for EL from the CSSL population. Long bars correspond to a short fragment of the interlocus chromosome, with black portions representing homozygous import fragments from ZYD00006 and slash stripes representing heterozygous import fragments from ZYD00006. The fragment distribution was imported based on the selected segment, and candidate EL-related genes were screened for within the 216 kb interval between markers BARCSOYSSR_08_0585 and BARCSOYSSR_08_0596.

The compositions of the individual genomes from 207 CSSL materials obtained through the repeated backcrossing of Suinong14 and ZYD00006 onto the Suinong 14 background for many years were analyzed to detect integrated fragments of the ZYD00006 genome. Given the variations in EL among these different population materials and the fact that EL values were significantly lower for the ZYD00006 variety as compared to the Suinong14 variety, the insertion of ZYD00006 genomic fragments was considered to be a potential contributing factor to reductions in EL. To test this possibility, 15 materials with extreme reductions in EL were selected for resequencing, using SSR markers combined with the chromosome 8 QTL interval identified above to evaluate the insertion region. A 216 kb insertion region (10.76–10.98 Mb) on chromosome 8 (BARCSOYSSR_08_0585 and BARCSOYSSR_08_0596) was identified as a candidate interval that may be associated with EL (Figure 2). One or more genes within this interval may thus serve as major regulators of EL.

### 2.4. RNA-Seq Analysis of Parental Epicotyl Tissues

**Figure 3:**
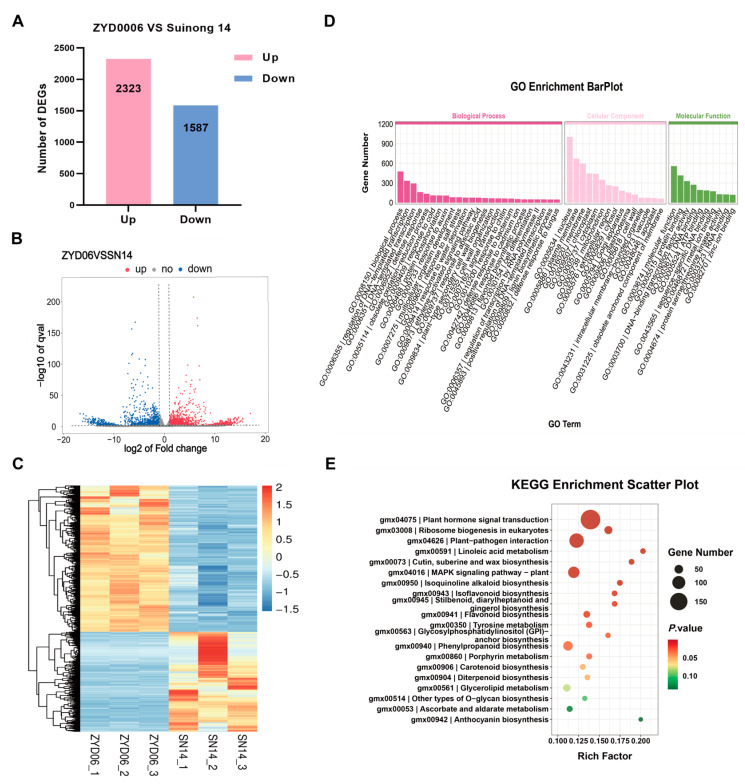
Transcriptomic analyses of Suinong 14 and ZYD00006 epicotyl tissues. (**A**) DEGs identified for this comparison. (**B**) Volcano plots and (**C**) heat map of the DEGs identified across transcriptome libraries. (**D**,**E**) GO and KEGG annotation results for identified DEGs.

To more reliably identify potential EL-related genes, RNA-Seq was next employed to screen for candidate genes within this target QTL interval. During the unfolding of the opposite true leaves, the epicotyl tissue situated between cotyledons and true leaves was harvested from the parental soybean varieties for RNA-Seq. In total, six RNA-Seq libraries were generated, yielding ~264 M of clean data, with a Q30% (the proportion of bases with quality value ≥ 30) ranging from 97.43% to 97.80% (Table S3). In total, 3910 differentially expressed genes (DEGs; |log2FC| ≥ 1, q < 0.05) were identified, of which 2323 and 1587 DEGs were, respectively, upregulated and downregulated in ZYD00006 as compared to SN14 (Figure 3A–C). GO enrichment analyses revealed that these DEGs were primarily related to defense responses (GO:0006952), DNA-templated transcription (GO:0006351) responses to auxin (GO:0009733), ethylene-activated signaling pathway activity (GO:0009873), multicellular organism development (GO:0007275), and flavonoid biosynthetic processes (GO:0009813). KEGG enrichment analyses further highlighted the enrichment of these DEGs in plant hormone signal transduction, isoflavonoid biosynthesis, flavone and flavonol biosynthesis, porphyrin metabolism, tyrosine metabolism, and other biological pathways (Figure 3D,E).

### 2.5. SNP Analyses of Candidate Genes within the QTL Interval

**Figure 4:**
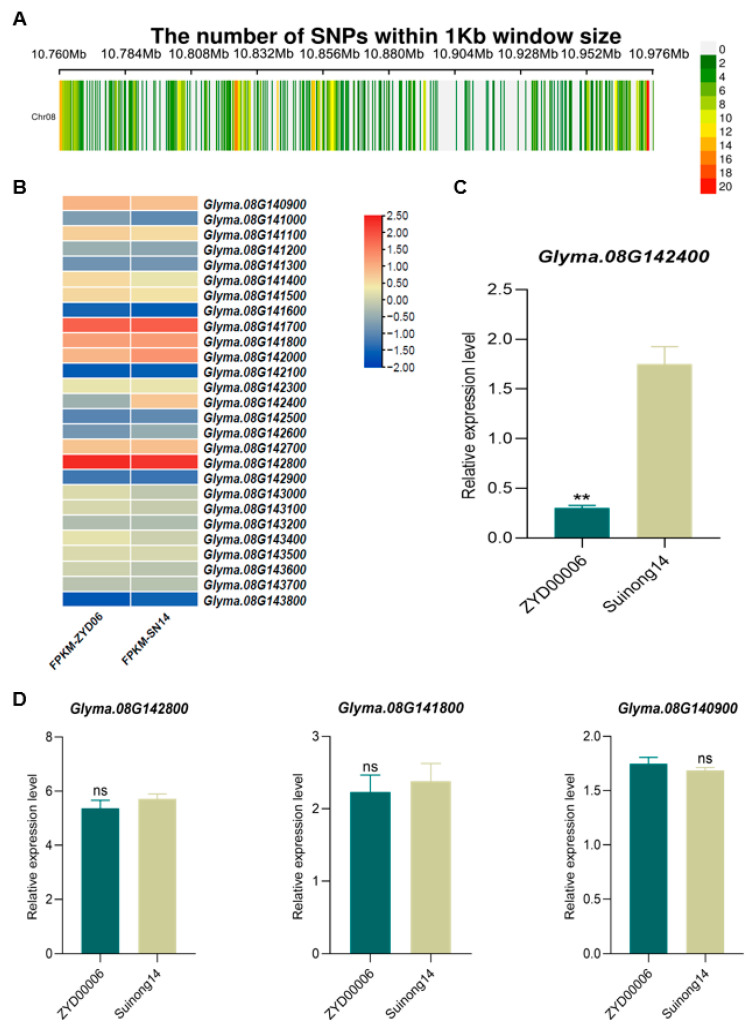
Identification of epicotyl development-related candidate genes. (**A**) SNP distributions within the target region of chromosome 8 in the Suinong 14 and ZYD00006. Entries on the right are the numbers of SNPs and Indels within each window in this region. (**B**) A heat map of gene expression associated with SNPs or Indels in this region. (**C**,**D**) Relative gene expression was analyzed via qPCR. Data were compared via Student’s *t*-tests (** *p* < 0.01; ns: not significant).

Using the Williams 82 reference genome, 31 genes were identified within the 216 kb target region of chromosome 8. Of these, 27 genes were identified harboring 263 SNPs and 87 Indels in this region in the Suinong14 and ZYD00006 reference genomes (Figure 4A). Of these, 45 SNPs were located within the exonic regions of 21 genes, while 218 SNPs were located within the promoter regions 3000 bp upstream of 25 genes, 4 Indels were located within exonic regions of 3 genes, and 83 Indels were located within the promoter regions 3000 bp upstream of 23 genes (Table S4). These results, together with the transcriptomic sequencing data, identified *Glyma.08G142400* as a gene that was significantly differentially expressed between epicotyl tissues from the Suinong14 and ZYD00006 varieties (Figure 4B). When genes from the transcriptomic dataset were selected for qPCR-based validation, only *Glyma.08G142400* was found to be expressed at significantly higher levels in Suinong14 epicotyl tissues as compared to those from ZYD00006 seedlings (Figure 4C,D and Figure S1). When comparing the Suinong 14 and ZYD00006 reference genomes, six SNPs and one Indel were identified within the *Glyma.08G142400* promoter region (Table S4). This suggests that these SNPs may account for the differential *Glyma.08G142400* expression evident in analyzed parental epicotyl tissues. As such, *Glyma.08G142400* was selected as a candidate gene for further analyses of EL development.

### 2.6. qPCR Analyses of Candidate Genes

**Figure 5:**
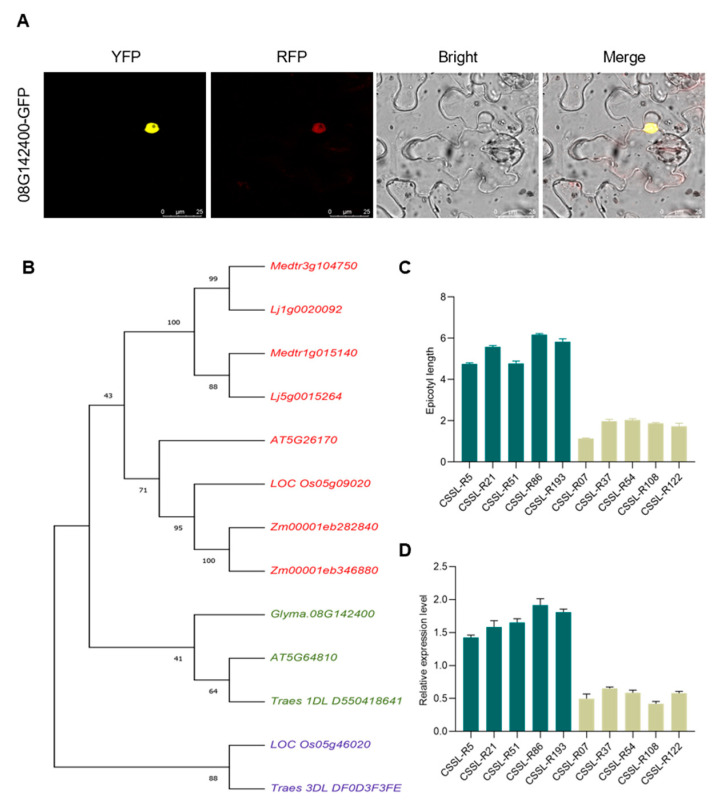
Analysis of *Glyma.08G142400* expression patterns in CSSL materials with differing EL. (**A**) Subcellular mapping results for *Glyma.08G142400*. (**B**) A phylogenetic tree-based analysis of *Glyma.08G142400* in six different plant species. (**C**) EL length measurements for 5 materials with long and 5 materials with short EL values were measured after full epicotyl extension. (**D**) The expression of *Glyma.08G142400* was analyzed in these 10 selected materials, with data corresponding to the mean results from three replicate measurements.

The identified candidate gene *Glyma.08G142400* encodes a member of the WRKY transcription factor family. Its CDS region was constructed into a eukaryotic expression vector containing a YFP tag, and the subcellular localization shows that Glyma.08G142400 is expressed in the nucleus. (Figure 5A). Phylogenetic analyses of *Glycine max* and five other species (*Arabidopsis thaliana*, *Medicago sativa*, *Lotus corniculatus*, *Zea may*, *Triticum aestivum*, and *Oryza sativa*) revealed that *Glyma.08G142400* and both *AT5G64810* and *Traes 1DL D550418641* were in the same branch and that they were closely related to one another (Figure 5B). Based on the phenotypic analyses of the CSSL population, 10 representative materials were collected, including 5 with relatively long EL (CSSL-R5, CSSL-R21, CSSL-R51, CSSL-R86, and CSSL-R193) and 5 with relatively short EL (CSSL-R07, CSSL-R37, CSSL-R54, CSSL-R108, and CSSL-R122). Analyses of gene expression in these materials revealed higher levels of *Glyma.08G142400* expression in the materials with longer EL as compared to those with shorter EL (Figure 5C,D), further supporting a potential role for *Glyma.08G142400* as a regulator of EL in the context of soybean development.

### 2.7. Haplotype Analyses of Glyma.08G142400

**Figure 6:**
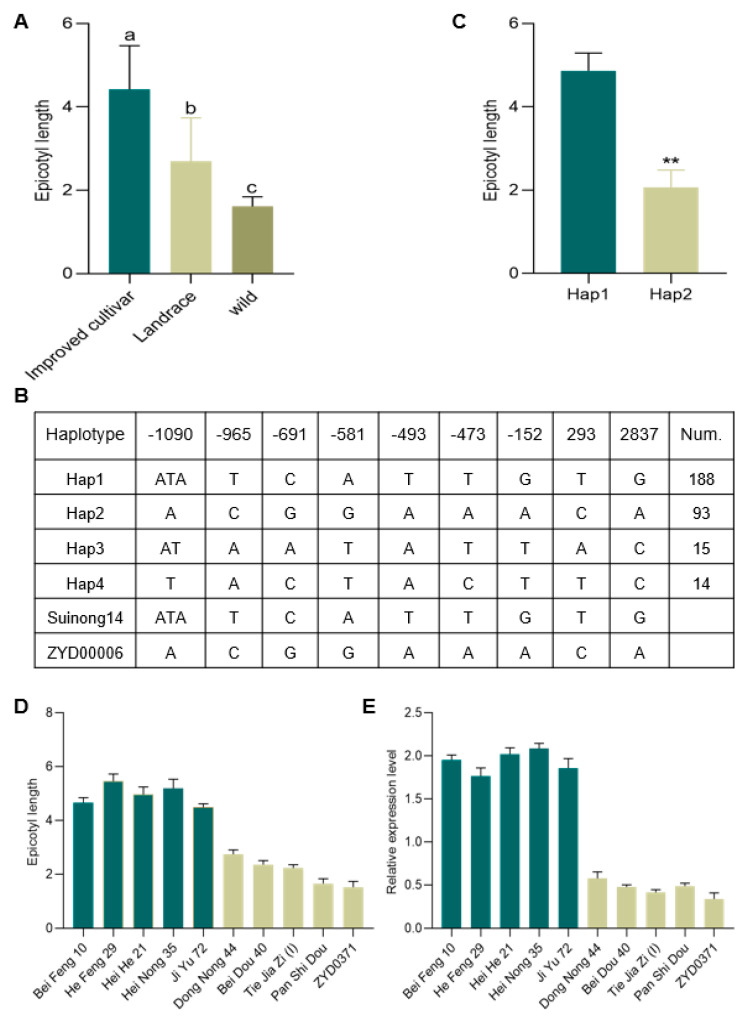
Haplotype analysis of *Glyma.08G142400*. (**A**) EL values for 310 natural soybean germplasm resources were analyzed with Duncan’s multiple range test. Significant differences (*p* < 0.05) are indicated with different letters. (**B**) Haplotype analysis of *Glyma.08G142400* based on the 310 analyzed soybean germplasm resources. (**C**) EL results from the superior Hap1 and Hap2 haplotypes were analyzed with Student’s *t*-tests (** *p* < 0.01). (**D**,**E**) EL measurements and relative *Glyma.08G142400* expression analyses were repeated three times for 10 Hap1 and Hap2 varieties.

To further confirm the potential role of *Glyma.08G142400* as a key regulator of soybean EL, we collected 310 germplasm resources (229 improved varieties, 71 local varieties, and 10 wild soybean varieties) from multiple regions for genome resequencing and EL measurements (Table S5). A statistical analysis of phenotypic data showed that the length of the epicotyl of the improved variety and landraces was higher than that of the wild soybean varieties, and the improved variety had the highest epicotyl height (Figure 6A). After combining the resequencing results and phenotypic data, haplotype analyses for *Glyma.08G142400* were performed with Dnasp5.0, ultimately leading to the identification of four total haplotypes among these 310 resources, of which two were superior haplotypes (>5% of the population) (Figure 6B). In total, eight SNPs and one Indel were identified when comparing the promoter and exonic regions of haplotype 1 (Hap1; including Suinong14) and Hap2 (including ZYD00006). Hap1 varieties exhibited significantly longer EL values as compared to Hap2 varieties (Figure 6C). When five Hap1 and five Hap2 varieties were selected to compare *Glyma.08G142400* expression in epicotyl samples, significantly higher levels of expression of this candidate gene were evident in Hap1 as compared to Hap2 varieties (Figure 6D,E). These haplotype analysis results therefore support a close relationship between *Glyma.08G142400* and EL during the early stages of soybean growth and development.

## 3. Discussion

Epicotyl length is a key agronomic trait of soybean plants, and it is influenced by genetic, planting, and environmental factors [3]. Appropriate planting depth and grain distribution density can positively affect EL, plant height, and soybean yields [5]. While it has been confirmed that genetics play a role in regulating EL [25], there has been little research to date focused on major epicotyl development-related genes. The use of CSSL populations to identify QTL intervals and the further mining of target genes via RNA-Seq can provide a robust and effective means of improving EL, thus supporting the breeding of soybean varieties that are more amenable to field planting using current technologies.

In the context of crop domestication and improvement efforts, certain superior alleles associated with improved traits of interest are inevitably selected, with variations in the associated genes impacting domestication and improvement outcomes. Current breeding efforts focus, in a large part, on identifying superior alleles as an approach to improving domestication rates for wild plant varieties so as to guide further crop improvement [26]. Over the course of domestication, rich soybean germplasm resources have been accumulated, including a wide range of wild, local, and extensively cultivated improved varieties [27]. Domestication inevitably results in the loss of genetic diversity among the resultant varieties as compared to wild plants, with the acquisition of desirable traits often coinciding with the loss of other potentially beneficial traits [26]. The genetic differences present in wild plants can thus serve as a resource for the breeding of new varieties of soybeans and other plants. Here, 310 natural soybean germplasm samples (10 wild soybean accessions, 71 landraces, and 229 improved soybean accessions) were employed to explore the relationship between EL and genotype. Relative to wild soybean accessions, EL values for the improved accessions were longer. The wild ZYD00006 variety and the improved Suinong14 variety were selected as parents to construct a CSSL population through extensive backcrossing, and the insertion sites of ZYD00006 fragments within the genome were then assessed through genomic resequencing guided by molecular markers [28]. Given the limited genomic interference for this genetic background, it was suitable for QTL identification. In this study, seven EL-related QTLs were identified, including a QTL interval of chromosome 8 that has been co-located for two years, as our next research focus. Of these, some overlapped with the hypocotyl development loci qHL-F [23] and with the seed germination loci qGRS-L [24] reported previously. This consistency highlights the accuracy of the QTLs identified in the present study.

Through analyses of ZYD00006 insertion fragments within the CSSL populations and the target QTL interval, RNA-Seq, and qPCR, *Glyma.08G142400* was ultimately screened as an EL-related gene encoding a 185 amino acid WRKY6 protein. The WRKY family is among the largest plant transcription factor families, with many WRKY proteins having been reported in species including soybean, rice, and *Arabidopsis* [29,30,31]. WRKY proteins include the WRKYGQK amino acid sequence together with a zinc finger motif, which is capable of binding to TTGAC(C/T) W-box cis-acting elements within the promoter regions of target genes [32]. WRKY has been widely reported to participate in various stages of plant growth and development. The *Arabidopsis* AtWRKY6 transcription factor is capable of functioning in concert with AtWRKY43, AtWRKY18, and AtWRKY60 to activate the expression of genes involved in abscisic acid (ABA) signaling and to thereby influence seed germination [33,34]. During the early stages of germination, the expression of *AtWRKY6* is decreased and *RAV1* expression is downregulated, influencing exogenous ABA hormone pathway activity. When *WRKY6* was overexpressed, *RAV1* expression was inhibited, and the progeny of positive plants showed ABA sensitivity during germination and seedling. During the development of Arabidopsis seeds, the expression level of *AtWRKY6* increases and affects the accumulation of fatty acids in the seeds. *WRKY* mutant plants exhibit an increase in fatty acid content and a significant increase in seed size [35]. This discovery reveals that WRKY6 can serve as a new resource for improving oil crop yield in molecular breeding. WRKY6 also plays a role in plant senescence. During leaf senescence, AtWRKY6 directly activates the promoter of SIRK, strongly inducing the expression of SIRK to participate in the regulation of leaf senescence [36]. A recent study of WRKY family proteins in soybean plants highlighted a role for GmWRKY6 and GmERF1 as joint regulators of soybean tolerance to low phosphorus stress [37]. These data offered confirmation of the ability of GmERF1 to interact with GmWRKY6 to inhibit phosphate transporter transcription, thereby impacting plant phosphorus uptake. Although there is no report on the involvement of wrky6 in regulating the development of epicotyls in soybean, it has been confirmed that WRKY family members play an important role in the photomorphogenesis of plant seedlings to affect the elongation of hypocotyls in Arabidopsis. It is reported that WRKY36 interacts with UV resistance locus 8 (uvr8) to inhibit the transcription of *HY5* and promote hypocotyl elongation [18]. Unlike AtWRKY36, WRKY32 negatively regulates hypocotyl length. The overexpression of *WRKY32* resulted in hypocotyl shortening in *Arabidopsis*, whereas knockout plants exhibited hypocotyl elongation. This is because AtCOP1 ubiquitylation of WRKY32 under dark conditions leads to its degradation, thus promoting the elongation of hypocotyl [38]. Here, *Glyma.08G142400* was identified as a WRKY transcription factor related to EL. The expression of this gene is evident during the epicotyl elongation and development stages, and analyses of *Glyma.08G142400* in soybean germplasm and CSSL populations revealed a close relationship between *Glyma.08G142400* haplotypes and EL. Analyses of the cis-acting elements within the *Glyma.08G142400* promoter region in the Suinong14 and ZYD00006 parental varieties revealed an additional L-box and an additional TATC-box motif within the promoter region of this gene in the Suinong14 genome as compared to the *ZYD00006* genome (Tables S6 and S7). The L-box is a photoresponsive element, while the TATC-box is involved in gibberellin (GA) responsiveness. Xiong et al. confirmed that the DELLA-ABI4-HY5 regulatory module is a novel molecular mechanism integrating gibberellin and the light signal antagonistic regulation of hypocotyl elongation [39]. Given the key role that GA plays in increasing auxin content and promoting accelerated cellular elongation [40], the differences in cis-acting elements within the *Glyma.08G142400* promoter between the Suinong14 and ZYD00006 varieties may at least partially account for differences in *Glyma.08G142400* expression in these parental lines. Overall, these results support the identification of *Glyma.08G142400* as a transcription factor that influences soybean EL, offering a foundation for the production of new soybean varieties that are better adapted to agricultural mechanization.

## 4. Materials and Methods

### 4.1. Plant Materials and Populations

CSSLs were constructed through crossing and backcrossing the wild ZYD00006 soybean variety and the cultivated Suinong14 soybean variety as the target population. The resultant CSSL populations were planted in a test field at the Xiangyang experimental farm of Northeast Agricultural University (45.58° N 126.92° E) in Harbin, with each material being replanted three times. Using a completely randomized design, each soybean germplasm was planted in the selected area with a length of 3 m, a row spacing of 35 cm, and a plant spacing of 20 cm. The mature seeds were harvested and planted in pots filled with peat soil to measure EL in greenhouse at 25 °C with a photoperiod of 16 h light and 8 h dark. In addition, 310 natural soybean germplasm resources were collected and planted in the same growth environment for EL measurements. Watering was performed with an appropriate amount of nutrient solution every day (Hoagland all nutrient solution, Cooler, Beijing, China). Three independent experiments were performed, and 10 plants of each germplasm were used to measure epicotyl length.

### 4.2. Soybean Epicotyl Length Measurement and Data Analysis

The distance between the cotyledon and the opposite true leaf was measured with Vernier calipers. EL measurements were made by selecting three plants at random and recording the mean EL value for analysis. Microsoft^®^Excel2016 was used to collate the data, Student’s *t*-test in GraphPad Prism 8 was used to test the significance, and Duncan’s multiple range test in SPSS 17.0 was used for multiple comparisons.

### 4.3. QTL Mapping

CSSLs were constructed using genetic maps published previously using the cultivated Suinong14 soybean variety and the wild ZYD00006 soybean variety [41]. EL-associated QTLs (LOD score > 3.0) were identified with WinQTL Cartographer 2.5 and composite interval mapping techniques.

### 4.4. ZYD00006 Chromosome Fragment Insertion Analysis

CSSL materials from the phenotypic extremes were selected for analyses of ZYD00006 chromosome fragment insertion sites based on CSSL genetic maps, with further screening of the major important fragment regions at major QTL sites.

### 4.5. SNP Analyses of Candidate Genes Associated with QTL Intervals

Major EL-related candidate QTLs were identified through QTL localization and ZYD00006 chromosome fragment insertion analysis. Candidate genes within these QTL regions were identified using the Williams 82 reference genome, with annotation using the available annotation information.

### 4.6. RNA-Seq Analyses

TRIzol was used as directed to extract RNA from soybean epicotyl samples, after which a Nanodrop ND-2000 instrument (Thermo Scientific, Waltham, MA, USA) was used to evaluate the A260/A280 ratio, while an Agilent Bioanalyzer 4150 system (Agilent Technologies, Santa Clara, CA, USA) was used to assess RNA integrity. The ABclonal mRNA-seq Lib Prep Kit (ABclonal, Wuhan, China) was used for paired-end library construction as directed. An Agilent Bioanalyzer 4150 system was used to examine library quality, after which an Illumina Novaseq 6000 instrument (Illumina, Shanghai, China) was used for sequencing, generating 150 bp paired-end reads.

Raw fastq format reads were initially processed using in-house Perl scripts, which removed adapter sequences and filtered out low-quality reads with an N ratio > 5%, yielding clean reads for subsequent analysis. These clean reads were separately aligned to the reference genome using HISAT2 (http://daehwankimlab.github.io/hisat2/, accessed on 2 November 2023), and read numbers mapped to each gene were then determined based on Feature Counts (http://subread.sourceforge.net/, accessed on 2 November 2023). FPKM values for all genes were calculated based on gene length and the number of reads mapped per gene, and differential gene expression was assessed with DESeq2 (http://bioconductor.org/packages/release/bioc/html/DESeq2.html, accessed on 2 November 2023), identifying DEGs as those with a |log2FC| > 1 and an adjusted *p* < 0.05.

### 4.7. qPCR

A qPCR approach was used to analyze the expression of candidate genes in the Suinong14 and ZYD00006 soybean varieties. Briefly, epicotyl samples were collected at specific time points, snap-frozen with liquid nitrogen, and total RNA was isolated with TRIzol. The PrimeScript™ RT kit (Takara Biotech Co., Beijing, China) was then used for cDNA preparation, and samples were then analyzed via qPCR with SuperReal PreMix Color (SYBR Green) (Tiangen Co., Beijing, China) and specific primers (Table S1). All analyses were independently repeated three times, and *Glyma.12g020500* served as a normalization control.

### 4.8. Subcellular Localization

*Agrobacterium tumefaciens* EHA105 carrying pEarlygate101-*Glyma.08G142400* was used to assess subcellular localization in young leaves of expanded *Nicotiana benthamiana*. The bacterial suspension was inoculated into tobacco leaves as described [21], and fluorescence signals were observed after 48 h using laser confocal microscopy (Leica, TCS SP8, Beijing, China).

### 4.9. Candidate Gene Haplotype Analyses

Candidate gene haplotypes were analyzed through the use of 310 natural soybean varieties. Candidate gene sequences were determined through genomic resequencing for these 310 varieties, which included analyses of the coding sequences and the associated promoter sequences (3000 bp upstream). SNPs within these regions were then identified through local BLAST analyses. All analyses were conducted with Haploview 4.2 (Cambridge, MA, USA) and the Haps format module, with correlations between EL and haplotypes being analyzed with GraphPad Prism 8.

## 5. Conclusions

In summary, EL-related QTLs were successfully identified in this study, using a CSSL population, on the cultivated Suinong14 soybean background containing fragments from the wild ZYD00006 soybean variety. In total, this approach revealed seven EL-related QTLs over a two-year period. Through whole-genome resequencing, chromosome insertion segment screening, RNA-seq, qPCR, and haplotype analyses, the candidate gene *Glyma.08G142400* was confirmed to be correlated with EL. These results offer a new foundation for efforts to explore the regulatory processes that govern epicotyl development, highlighting avenues for further mechanistic research.

## Figures and Tables

**Table 1:** The EL of CSSL populations.

Trait	Year	Parents		CSSL Population (n = 207)		
		ZYD00006	Suinong14	Mean ± SD ^1^	Kurtosis ^2^	Skewness ^3^
EL	2021	1.27 **	4.99	4.06 ± 1.06	0.27	−0.84
	2022	1.25 **	5.14	4.04 ± 1.1	0.34	−0.89

Note: ** indicates *p* ≤ 0.01, ^1^ standard deviation, ^2^ a statistic that describes the steepness of the distribution of all values of a variable, and ^3^ a statistic that describes the symmetry of a variable’s value distribution.

**Table 2:** Identification of QTL loci associated with EL in CSSL population.

Trait	Year	Chr/LG ^a^	QTL	Position (Mb)	LOD ^b^	R^2 c^	ADD ^d^	Previous Research Reports
EL	2021	Chr03/N	*qEL21-03*	34.7	3.5	4.2	−0.07	
		Chr17/D2	*qEL21-17*	21.9	11.1	5.7	−1.32	
		Chr08/A2	*qEL21-08*	10.6	8.4	6	0.05	
		Chr13/F	*qEL21-13*	10.2	7.3	6.5	0.26	*qHL-F* [23]
	2022	Chr12/H	*qEL22-12*	22.6	4.5	3.2	0.33	
		Chr08/A2	*qEL22-08*	10.6	7.7	7.8	0.12	
		Chr19/L	*qEL22-19*	44.9	6.3	4.4	−1.18	*qGRS-L* [24]
		Chr03/N	*qEL22-03*	31.5	3.4	2.8	−0.03	

Note: ^a^ chromosome number and linkage group, ^b^ logarithm of odds ratio, ^c^ phenotypic variance explained, and ^d^ additive effects value.

## Data Availability

The data presented in this study are available on request from the corresponding author.

## Supplementary Materials

The supporting information can be downloaded at https://www.mdpi.com/article/10.3390/ijms25063296/s1.
