# Supplementary material for: QTL Mapping and Data Mining to Identify Genes Associated with Soybean Epicotyl Length Using Cultivated Soybean and Wild Soybean
Source: Int J Mol Sci. 2024 Mar 14;25(6):3296. doi: 10.3390/ijms25063296 (PMC10970105; doi:10.3390/ijms25063296)
Supplement: Supplementary file 1 [file ijms-25-03296-s001.zip › Figure S1.pdf]

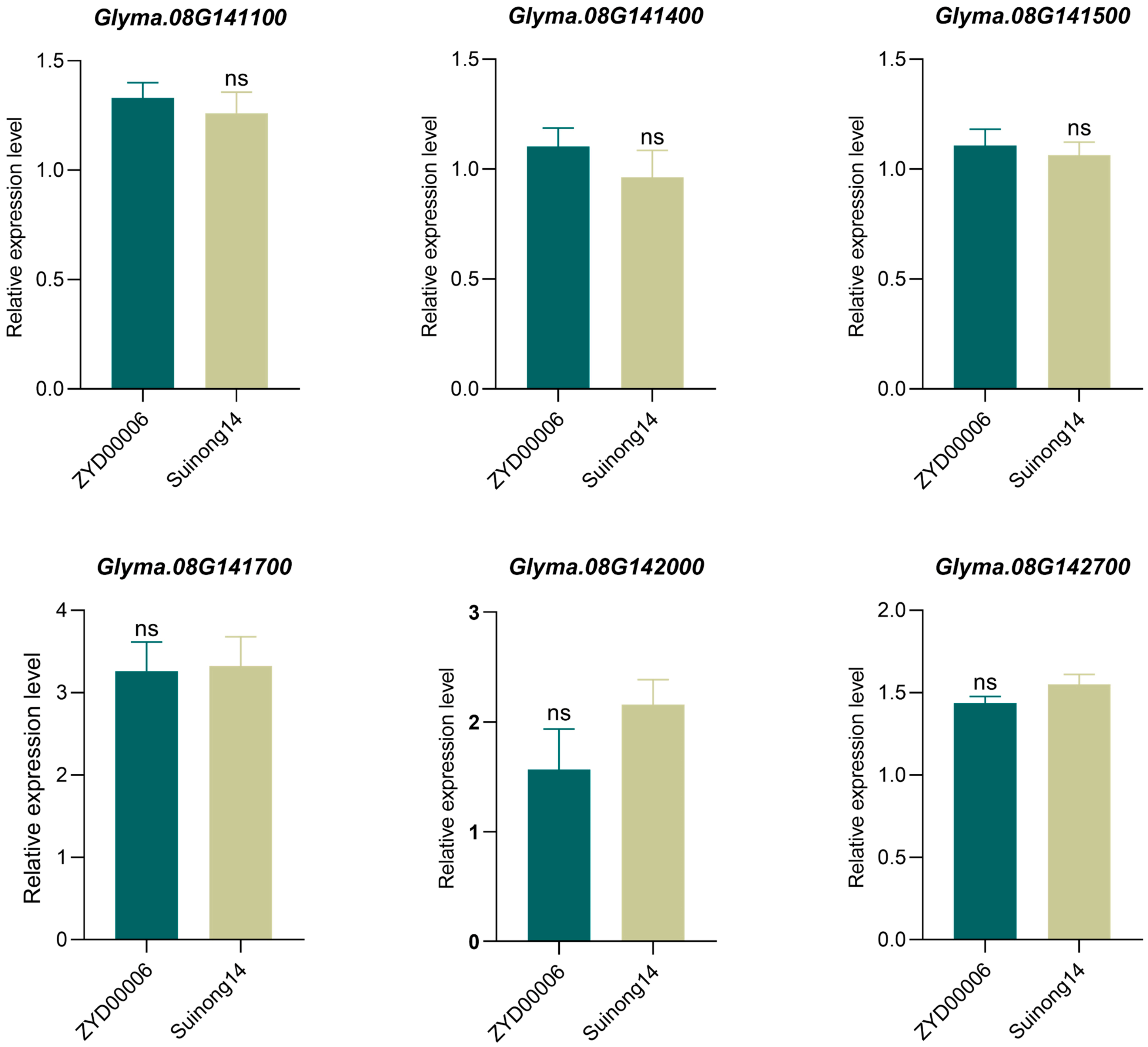

Figure S1: Relative gene expression in candidate was analyzed via qPCR. Data were compared via Student's *t*-tests (ns: not significant).
